# Focusing Bistatic Forward-Looking Synthetic Aperture Radar Based on an Improved Hyperbolic Range Model and a Modified Omega-K Algorithm

**DOI:** 10.3390/s19173792

**Published:** 2019-09-01

**Authors:** Chenchen Wang, Weimin Su, Hong Gu, Jianchao Yang

**Affiliations:** School of Electronic and Optical Engineering, Nanjing University of Science and Technology, Nanjing 210094, China

**Keywords:** bistatic synthetic aperture radar (SAR), hyperbolic approximation, phase compensation, modified omega-K

## Abstract

For parallel bistatic forward-looking synthetic aperture radar (SAR) imaging, the instantaneous slant range is a double-square-root expression due to the separate transmitter-receiver system form. The hyperbolic approximation provides a feasible solution to convert the dual square-root expression into a single-square-root expression. However, some high-order terms of the range Taylor expansion have not been considered during the slant range approximation procedure in existing methods, and therefore, inaccurate phase compensation occurs. To obtain a more accurate compensation result, an improved hyperbolic approximation range form with high-order terms is proposed. Then, a modified omega-K algorithm based on the new slant range form is adopted for parallel bistatic forward-looking SAR imaging. Several simulation results validate the effectiveness of the proposed imaging algorithm.

## 1. Introduction

Synthetic aperture radar (SAR) attracts massive research enthusiasm among researchers due to its excellent ability to detect targets without the limitation of the external environment [1]. The penetration ability of SAR makes it irreplaceable compared with optical imaging, while it is challenging in traditional monostatic SAR to obtain excellent imaging performance in forward-looking imaging mode, which limits the application of SAR technology. To solve the problem, bistatic SAR has been widely used for forward-looking imaging due to its particular system configuration. The separate transmitter and receiver configuration provides extra advantages like reliable hiding power and system flexibility [2].

One-stationary bistatic SAR, as a special form of general bistatic SAR, was first studied for forward-looking imaging. Several methods have been proposed, such as the squint minimization [3,4], the keystone transform [5], and the ellipse model [2,4]. The Doppler frequency is decided by the moving transmitter or the moving receiver, which is similar to monostatic SAR. Then, the bistatic SAR was proposed where both the transmitter and the receiver are moving. The azimuth resolution is determined by both platforms. For bistatic forward-looking SAR, the difficulty of imaging algorithms lies in the solution of the two-dimensional spectrum because of its unique double-square-root form of echo signal expression [6,7]. Some basic studies of bistatic SAR were proposed to illustrate the advantages [6,8]. Compared with the monostatic situation, the principle of stationary phase (POSP) cannot be applied to solve the derivative zero point when performing azimuth Fourier transform. Several methods have been proposed to solve the problem. Loffeld’s bistatic formula (LBF) was proposed to solve the double-square-root expression [6]. Respective stationary points of the transmitter and receiver are obtained first to transform the double-square-root expression into Taylor expansion form. Then, the ultimate spectrum is solved based on the joint stationary point of the Taylor expression. The contributions of the transmitter and receiver are assumed to be the same, which leads to approximation errors. The extended Loffeld’s bistatic formula (ELBF) [9] and the modified Loffeld’s bistatic formula (MLBF) [10] were proposed later to improve the solution process of stationary points. These two methods assign different weights on the transmitter and receiver. However, all three LBF methods need to solve the stationary points three times, which leads to deduction complexity. The method of series reversion (MSR) [11] is a widely-used method for precisely solving those equations with series terms. In SAR imaging algorithms, Taylor expansion is regarded as a common operation, and MSR can be applied to solve the Fourier transform composed of Taylor expansion. However, it is still challenging to conduct imaging algorithm deduction due to the series form.

To simplify the solution of the spectrum, the hyperbolic approximation was utilized to transform the echo expression with the double-square-root form into the expression with the single-square-root form. In the first version, a parameter named the equivalent speed was defined [12]. In the improved version, two more parameters (the equivalent slant range and the equivalent squint angle) [13] were added in the hyperbolic function to approximate the range more accurately. Moreover, an improved hyperbolic approximation model with additional parameters was proposed for residual compensation [14]. However, considering the solution process in the methods mentioned above, the defined parameters are solved by setting the constant term, the linear term, and the quadratic term of the Taylor expansion of echo equal, which means the influence of the cubic term, the quartic term, and the remaining terms is ignored. In this article, we propose a new model to finish the hyperbolic approximation.

As for imaging algorithms, range Doppler (RD) imaging algorithms, chirp scaling (CS) imaging algorithms, back-projection (BP) imaging algorithms, and omega-K imaging algorithms based on the LBF spectrum, the MSR spectrum, and the hyperbolic approximation spectrum have been proposed in the past few years [9,14,15,16]. For RD imaging algorithms, it is too fundamental to handle the complex situation of bistatic SAR system. The calculation time consumption is a severe problem for real-time processing when applying BP imaging algorithms. For CS imaging algorithms, it is difficult for researchers to conduct formula derivation. Thus, the omega-K imaging algorithm is selected in this article to finish imaging.

To approximate the slant range more accurately, the cubic term and the quartic term are taken into account in this article. An equivalent hyperbolic range model is introduced first to lay the foundation of the imaging algorithm. The range error analysis is provided to demonstrate the approximation ability of the proposed range model immediately. Then, the modified omega-K imaging algorithm including the signal model and detailed processing steps are presented. Finally, some experimental simulations are given to prove the efficiency of the proposed algorithm.

This article is organized as follows. Section 2 gives the geometry of the bistatic forward-looking SAR and the equivalent hyperbolic range model corresponding to the bistatic system. Section 3 gives the detailed modified omega-K imaging algorithm. Simulation results are given in Section 4 to validate the proposed algorithm. Section 5 provides the conclusion.

## 2. Geometry and Equivalent Slant Range Model

The parallel bistatic forward-looking SAR system diagram in the Cartesian coordinate system and the derived equivalent slant range model are established first. Then, the analysis of range error based on the equivalent slant range model is provided.

### 2.1. Equivalent Slant Range Model

**Figure 1:**
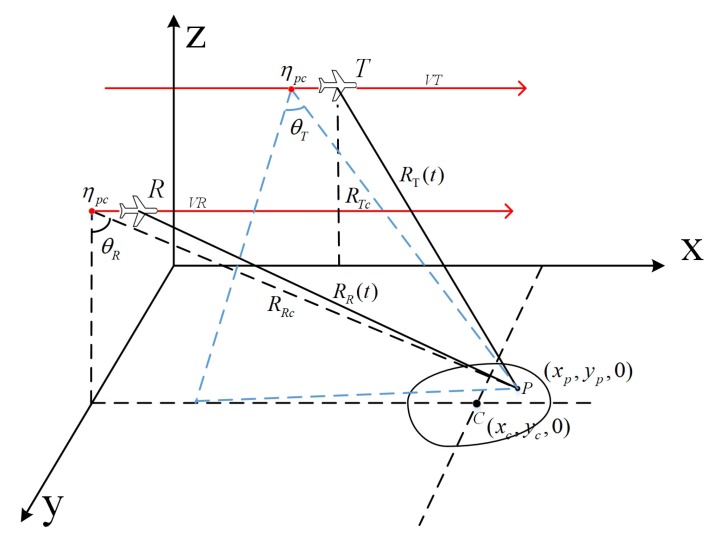
Geometry of forward-looking bistatic SAR.

Figure 1 shows the geometry of parallel bistatic forward-looking SAR. The transmitter *T* and the receiver *R*move along the parallel red lines parallel to the *x*-axis. $\eta_{pc}$ is the synthetic aperture center time of the imaging scene. $\left(x_{c},y_{c},0\right)$ is the location coordinate of the imaging center, and $\left(x_{p},y_{p},0\right)$ is the location of an arbitrary target *P* in the imaging scene. $R_{Tc}$ is the slant range between the transmitter and the target *P* at the phase center crossing time $\eta_{pc}$, and $R_{Rc}$ is the range between the receiver and the target *P* at $\eta_{pc}$. The approximate forward-looking angle of the receiver is $\theta_{R}$, and the approximate squint angle of the transmitter is $\theta_{T}$. $V_{T}$ and $V_{R}$ represent the speed of the transmitter and the receiver, respectively. It is assumed that both the transmitter and the receiver can cover the imaging scene during the aperture synthesis.

The instantaneous slant ranges from the transmitter and the receiver to the target *P* are:

(1) $$\begin{array}{c} R_{T}\left(\eta \right)=\sqrt{R_{Tc}^{2}+V_{T}^{2}{\left(\eta -\eta_{pc}\right)}^{2}-2R_{Tc}V_{T}\left(\eta -\eta_{pc}\right)\sin\theta_{T}},\\ R_{R}\left(\eta \right)=\sqrt{R_{Rc}^{2}+V_{R}^{2}{\left(\eta -\eta_{pc}\right)}^{2}-2R_{Rc}V_{R}\left(\eta -\eta_{pc}\right)\sin\theta_{R}}, \end{array}$$

where η is the slow time.

Thus, the total range is:

(2) $$R\left(\eta \right)=R_{T}\left(\eta \right)+R_{R}\left(\eta \right).$$

It is challenging to solve the two-dimensional spectrum due to the double-square-root expression form of $R\left(\eta \right)$. The hyperbolic approximation [12] can be used to convert the double-square-root form to a single-square-root form by defining the equivalent speed and equivalent angle. Traditional hyperbolic approximation [12,13,14] ignored the high-order terms of the Taylor expansion of $R\left(\eta \right)$. To realize a more accurate compensation, an improved equivalent slant range with high-order terms is proposed. The range model is expressed as:

(3) $$R_{e}\left(\eta \right)=\sqrt{R_{e}^{2}+V_{e}^{2}{\left(\eta -\eta_{pc}\right)}^{2}-2R_{e}V_{e}\left(\eta -\eta_{pc}\right)\sin\theta_{e}}+E{\left(\eta -\eta_{pc}\right)}^{3}+F{\left(\eta -\eta_{pc}\right)}^{4},$$

(4) $$R\left(\eta \right)=2R_{e}\left(\eta \right),$$

where $R_{e}$, $V_{e}$, and $\theta_{e}$ are the new equivalent slant range at phase crossing time, the new equivalent speed, and the new equivalent squint angle. Compared with existing hyperbolic approximation algorithms, the proposed range model adds two additional high-order terms for range error compensation. To solve the unknown variables, we first expand Equations (1) and (3) into a fourth-order Taylor series at $\eta =\eta_{pc}$. Then, we get:

(5) $$\begin{array}{cc} R_{T}\left(\eta \right)= & R_{Tc}-V_{T}\sin\theta_{T}\left(\eta -\eta_{pc}\right)+\frac{V_{T}^{2}\cos\theta_{T}^{2}}{2R_{Tc}}{\left(\eta -\eta_{pc}\right)}^{2}+\\ \; & \frac{V_{T}^{3}\sin\theta_{T}\cos^{2}\theta_{T}}{2R_{Tc}^{2}}{\left(\eta -\eta_{pc}\right)}^{3}+\frac{V_{T}^{4}\cos^{2}\theta_{T}\left(5\sin^{2}\theta_{T}-1\right)}{8R_{Tc}^{3}}{\left(\eta -\eta_{pc}\right)}^{4}, \end{array}$$

(6) $$\begin{array}{cc} R_{R}\left(\eta \right)= & R_{Rc}-V_{R}\sin\theta_{R}\left(\eta -\eta_{pc}\right)+\frac{V_{R}^{2}\cos\theta_{R}^{2}}{2R_{Rc}}{\left(\eta -\eta_{pc}\right)}^{2}+\\ \; & \frac{V_{R}^{3}\sin\theta_{R}\cos^{2}\theta_{R}}{2R_{Rc}^{2}}{\left(\eta -\eta_{pc}\right)}^{3}+\frac{V_{R}^{4}\cos^{2}\theta_{R}\left(5\sin^{2}\theta_{R}-1\right)}{8R_{Rc}^{3}}{\left(\eta -\eta_{pc}\right)}^{4}, \end{array}$$

(7) $$\begin{array}{cc} R_{e}\left(\eta \right)= & R_{e}-V_{e}\sin\theta_{e}\left(\eta -\eta_{pc}\right)+\frac{V_{e}^{2}\cos\theta_{e}^{2}}{2R_{e}}{\left(\eta -\eta_{pc}\right)}^{2}+\\ \; & \frac{V_{e}^{3}\sin\theta_{e}\cos^{2}\theta_{e}}{2R_{e}^{2}}{\left(\eta -\eta_{pc}\right)}^{3}+\frac{V_{e}^{4}\cos^{2}\theta_{e}\left(5\sin^{2}\theta_{e}-1\right)}{8R_{e}^{3}}{\left(\eta -\eta_{pc}\right)}^{4}+\\ \; & E{\left(\eta -\eta_{pc}\right)}^{3}+F{\left(\eta -\eta_{pc}\right)}^{4}. \end{array}$$

Substituting Equations (5)–(7) into Equations (2) and (4) and letting the first five terms of Taylor expansion be equal, then we get:

(8) $$\left\{\begin{array}{c} R_{Tc}+R_{Rc}=2R_{e}\\ V_{T}\sin\theta_{T}+V_{R}\sin\theta_{R}=2V_{e}\sin\theta_{e}\\ \frac{V_{T}^{2}\cos\theta_{T}^{2}}{2R_{Tc}}+\frac{V_{R}^{2}\cos\theta_{R}^{2}}{2R_{Rc}}=2\frac{V_{e}^{2}\cos\theta_{e}^{2}}{2R_{e}}\\ \frac{V_{T}^{3}\sin\theta_{T}\cos^{2}\theta_{T}}{2R_{Tc}^{2}}+\frac{V_{R}^{3}\sin\theta_{R}\cos^{2}\theta_{R}}{2R_{Rc}^{2}}=2\left(\frac{V_{e}^{3}\sin\theta_{e}\cos^{2}\theta_{e}}{2R_{e}^{2}}+E\right)\\ \frac{V_{T}^{4}\cos^{2}\theta_{T}\left(5\sin^{2}\theta_{T}-1\right)}{8R_{Tc}^{3}}+\frac{V_{R}^{4}\cos^{2}\theta_{R}\left(5\sin^{2}\theta_{R}-1\right)}{8R_{Rc}^{3}}=2\left[\frac{V_{e}^{4}\cos^{2}\theta_{e}\left(5\sin^{2}\theta_{e}-1\right)}{8R_{e}^{3}}+F\right]. \end{array}\right.$$

Solving the five equations in Equation (8), then we get:

(9) $$\left\{\begin{array}{c} R_{e}=\frac{1}{2}\left(R_{Tc}+R_{Rc}\right)\\ V_{e}=\sqrt{A^{2}+B}\\ \theta_{e}=\arcsin\left(A/V_{e}\right)\\ E=C-\frac{V_{e}^{3}\sin\theta_{e}\cos^{2}\theta_{e}}{2R_{e}^{2}}\\ F=D-\frac{V_{e}^{4}\cos^{2}\theta_{e}\left(5\sin^{2}\theta_{e}-1\right)}{8R_{e}^{3}}, \end{array}\right.$$

where:

(10) $$\left\{\begin{array}{c} A=\left(V_{T}\sin\theta_{T}+V_{R}\sin\theta_{R}\right)/2\\ B=\left(\frac{V_{T}^{2}\cos^{2}\theta_{T}}{R_{Tc}}+\frac{V_{R}^{2}\cos^{2}\theta_{R}}{R_{Rc}}\right)R_{e}/2\\ C=\frac{V_{T}^{3}\sin\theta_{T}\cos^{2}\theta_{T}}{4R_{Tc}^{2}}+\frac{V_{R}^{3}\sin\theta_{R}\cos^{2}\theta_{R}}{4R_{Rc}^{2}}\\ D=\frac{V_{T}^{4}\cos^{2}\theta_{T}\left(5\sin^{2}\theta_{T}-1\right)}{16R_{Tc}^{2}}+\frac{V_{R}^{4}\cos^{2}\theta_{R}\left(5\sin^{2}\theta_{R}-1\right)}{16R_{Rc}^{2}}. \end{array}\right.$$

At this point, all defined variables are solved. The range error analysis based on the new equivalent range model is presented next.

### 2.2. Range Error Analysis

**Figure 2:**
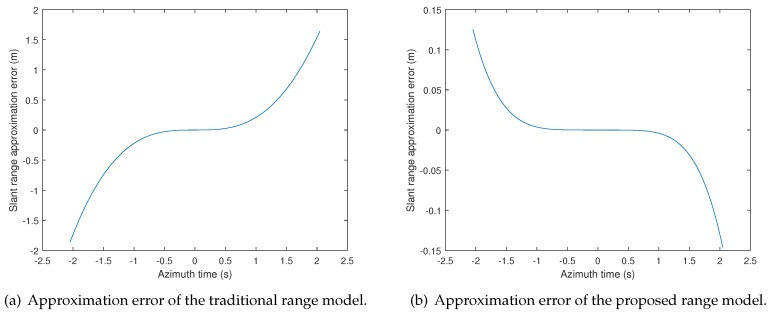
Approximation error of the bistatic slant range. (**a**) Approximation error of the traditional range model. (**b**) Approximation error of the proposed range model.

To evaluate the proposed equivalent range model, an analysis of the range error based on an X-band bistatic SAR system is given. The simulated parameters are listed in Table 1. The results of the equivalent hyperbolic slant range error are shown in Figure 2.

Figure 2a is the approximation error of the traditional hyperbolic approximation range model [13], where the high-order terms are ignored. Figure 2b is the approximation error of the proposed hyperbolic range model. The constant term, the linear term, and the quadratic term in Equations (5)–(7) are used to solve the defined variables. Thus, the residual terms lead to the approximation slant range error. To prove that the proposed model can reduce the range error compared with the traditional model, we first give the expression of the traditional model and its corresponding Taylor expansion, which are:

(11) $$R_{t}\left(\eta \right)=\sqrt{R_{t}^{2}+V_{t}^{2}{\left(\eta -\eta_{pc}\right)}^{2}-2R_{t}V_{e}\left(\eta -\eta_{pc}\right)\sin\theta_{t}},$$

(12) $$\begin{array}{cc} R_{t}\left(\eta \right)= & R_{t}-V_{t}\sin\theta_{t}\left(\eta -\eta_{pc}\right)+\frac{V_{t}^{2}\cos\theta_{t}^{2}}{2R_{t}}{\left(\eta -\eta_{pc}\right)}^{2}+\\ \; & \frac{V_{t}^{3}\sin\theta_{t}\cos^{2}\theta_{t}}{2R_{t}^{2}}{\left(\eta -\eta_{pc}\right)}^{3}+\frac{V_{t}^{4}\cos^{2}\theta_{t}\left(5\sin^{2}\theta_{t}-1\right)}{8R_{t}^{3}}{\left(\eta -\eta_{pc}\right)}^{4} \end{array}$$

where $R_{t}\left(\eta \right)$, $R_{t}$, $V_{t}$, and $\theta_{t}$ are the variables in traditional range model. The error in Figure 2a is the difference between the sum of the cubic terms, the quartic terms, and the residual terms in Equations (5) and (6) and the sum of the cubic term, the quartic term, and the residual term in Equation (12), while the error in Figure 2b is the difference between the sum of the residual terms in Equations (5) and (6) and the residual term in Equation (7). The error caused by the cubic and quartic terms is eliminated. From Figure 2, it can be found that the error in Figure 2a is up to 1.9 m, while the error in Figure 2b is less than 0.15 m. According to the parameters listed in Table 1, the approximation slant range error of the proposed model is much less than a range solution cell. Therefore, the proposed equivalent slant range model is more accurate than the traditional range model. The following imaging algorithm is derived based on the proposed range model.

## 3. Imaging Algorithm

According to the previous analysis, the improved hyperbolic approximation model can equal the true slant range better than traditional hyperbolic approximate models. In this section, a modified omega-K algorithm based on the improved equivalent range model is proposed for the parallel bistatic forward-looking SAR imaging.

### 3.1. Signal Model

Assume that a linear frequency-modulated signal is transmitted from the transmitter to the receiver. Then, the base-band echo signal of an arbitrary target *P* is given as:

(13) $$S_{1}\left(t_{r},\eta \right)=\exp\left\{j\pi \gamma \left[t_{r}-\frac{2R_{e}\left(\eta \right)}{c}\right]\right\}\exp\left[-j\frac{4\pi R_{e}\left(\eta \right)}{\lambda }\right]$$

where γ is the range chirp rate, c is the light speed, λ is the wavelength, $t_{r}$ is the fast time, and η is the slow time. To simplify the expression and further derivation, the envelopes of the range and azimuth are ignored.

Transforming Equation (13) into the range-frequency azimuth-time domain yields:

(14) $$S_{2}\left(f_{r},\eta \right)=\exp\left(-j\frac{\pi f_{r}^{2}}{\gamma }\right)\exp\left[-j\frac{4\pi \left(f_{r}+f_{c}\right)}{c}R_{e}\left(\eta \right)\right]$$

where $f_{r}$ is the frequency domain variable corresponding to $t_{r}$ and $f_{c}$ is the carrier frequency. From Equation (14), it can be easily found that the first exponential term is the range frequency modulation term. This term can be compensated by multiplying its complex conjugate in the range frequency domain. Thus, the first frequency modulation compensation function is:

(15) $$H_{1FM}\left(f_{r},\eta \right)=\exp\left(j\frac{\pi f_{r}^{2}}{\gamma }\right).$$

Multiplying Equation (14) by Equation (15) yields:

(16) $$S_{3}\left(f_{r},\eta \right)=\exp\left[-j\frac{4\pi \left(f_{r}+f_{c}\right)}{c}R_{e}\left(\eta \right)\right].$$

The exponential term in Equation (16) indicates the severe coupling between range and azimuth. To finish the phase focusing, a modified omega-K algorithm based on the signal model is presented.

### 3.2. Modified Omega-K Imaging Algorithm

To analyze the exponential term in Equation (16), Equation (3) is substituted into Equation (16) firstly. Then, we get:

(17) S4fr,η=exp−j4πfr+fcc[Re2+Ve2η−ηpc2−2ReVeη−ηpcsinθe=+Eη−ηpc3+Fη−ηpc4].

Equation (17) shows that the signal consists of the traditional hyperbolic term and high-order terms. The traditional omega-K can handle the hyperbolic term well, but cannot handle the high-order terms. The first step of the omega-K algorithm is the compensation of the cubic term and the quartic term. Variable substitution is performed on Equation (17), and then, we get:

(18) S5kr,X=exp−jkrRe2+X−Xpc2−2ReX−Xpcsinθe=+EVe3X−Xpc3+FVe4X−Xpc4]

where $k_{r}=\frac{4\pi \left(f_{r}+f_{c}\right)}{c}$ is the wavenumber, $X=V_{e}\eta$, and $X_{pc}=V_{e}\eta_{pc}$. Then, we get $R_{e}\left(\eta \right)=R_{e}\left(X\right)$.

Transforming Equation (18) into two-dimensional wavenumber domain yields:

(19) $$\begin{array}{cc} S_{6}\left(k_{r},k_{x}\right) & =\int S_{5}\left(k_{r},X\right)\exp\left(-jk_{x}X\right)dX\\ \; & =\int \exp\left\{-jk_{r}R_{e}\left(X\right)\right\}\exp\left(-jk_{x}X\right)dX\\ \; & =\int \exp\left\{-j\phi \left(k_{r},k_{x},X\right)\right\}dX \end{array}$$

where $k_{x}=\frac{2\pi f_{a}}{V_{e}}$, $f_{a}$ is the azimuth frequency, and:

(20) $$\begin{array}{cc} \phi \left(k_{r},k_{x},X\right)= & k_{r}\left[\sqrt{R_{e}^{2}+{\left(X-X_{pc}\right)}^{2}-2R_{e}\left(X-X_{pc}\right)\sin\theta_{e}}\right.\\ \; & \left.+\frac{E}{V_{e}^{3}}{\left(X-X_{pc}\right)}^{3}+\frac{F}{V_{e}^{4}}{\left(X-X_{pc}\right)}^{4}\right]+k_{x}X. \end{array}$$

**Figure 3:**
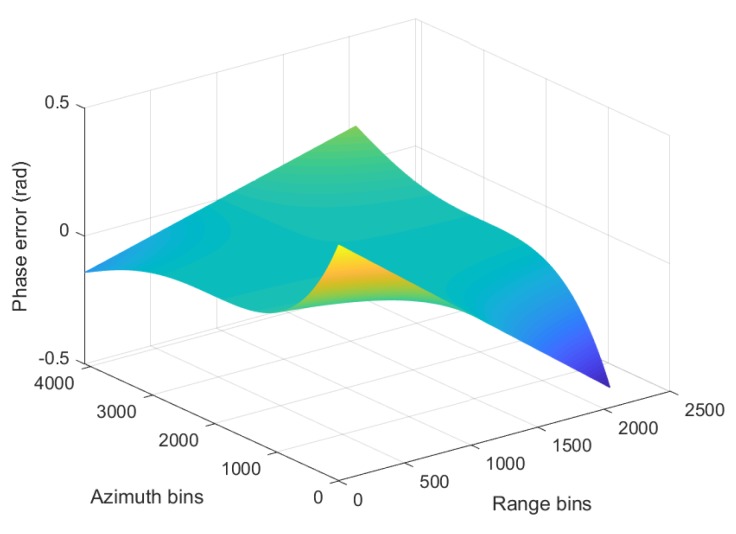
Phase error simulation.

To solve Equation (19), the stationary phase point of $\phi \left(k_{r},k_{x},X\right)$ should be obtained firstly. However, the existence of high-order terms complicates the solution process. For further analysis, the phase is first rewritten as:

(21) $$\phi \left(k_{r},k_{x},X\right)=\phi_{t}\left(k_{r},k_{x},X\right)+k_{r}\left[\frac{E}{V_{e}^{3}}{\left(X-X_{pc}\right)}^{3}+\frac{F}{V_{e}^{4}}{\left(X-X_{pc}\right)}^{4}\right]$$

where $\phi_{t}\left(k_{r},k_{x},X\right)$ is the traditional phase term. It is widely accepted that if the phase error is smaller than π/4 [1], the imaging performance will not be affected much by the approximation. The phase error simulation is given in Figure 3.

From Figure 3, it can been seen that all absolute phase errors are less than π/4. Thus, the stationary phase point of $\phi_{t}\left(k_{r},k_{x},X\right)$ is regarded as the approximate stationary phase point of $\phi \left(k_{r},k_{x},X\right)$. The approximate stationary phase point of $\phi \left(k_{r},k_{x},X\right)$ is:

(22) $$X^{*}=-\frac{k_{x}R_{e}\sin\theta_{e}}{\sqrt{k_{r}^{2}-k_{x}^{2}}}+R_{e}\sin\theta_{e}+X_{pc},$$

where $X^{*}$ is only a designation of the solution and (*) is not an operator.

Substituting Equation (22) in Equation (19) and applying POSP yield the two-dimensional wavenumber domain signal as:

(23) S7kr,kx=exp−jkr2−kx2Recosθe−jkxResinθe+Xpc=−jkrEVe3X*−Xpc3+FVe4X*−Xpc4.

The cubic term and quartic term in Equation (23) can be easily compensated by multiplying its conjugate form. Therefore, the high-order filter is:

(24) $$H_{2}\left(k_{r},k_{x}\right)=\exp\left\{jk_{r}\left[\frac{E}{V_{e}^{3}}{\left(X^{**}-X_{pc}\right)}^{3}+\frac{F}{V_{e}^{4}}{\left(X^{**}-X_{pc}\right)}^{4}\right]\right\}$$

where $X^{**}$ is the value of $X^{*}$ at the reference range and (**) is not an operator.

Multiplying Equations (23) and (24), we get the compensated signal for the further omega-K imaging algorithm. The signal is:

(25) $$S_{8}\left(k_{r},k_{x}\right)=\exp\left\{-j\sqrt{k_{r}^{2}-k_{x}^{2}}R_{e}\cos\theta_{e}-jk_{x}\left(R_{e}\sin\theta_{e}+X_{pc}\right)\right\}.$$

A two-step omega-K is performed on Equation (25) to finish the imaging focusing.

The first step is the bulk focusing. A reference function is designed based on the reference range to finish coarse focusing. This filter can compensate the phase of signals of those points at the reference range. The reference function is:

(26) $$H_{rf}\left(k_{r},k_{x}\right)=\exp\left\{j\sqrt{k_{r}^{2}-k_{x}^{2}}R_{ref}\cos\theta_{e}+jk_{x}\left(R_{ref}\sin\theta_{e}+X_{pc}\right)\right\}.$$

Multiplying Equations (25) and (26) gets:

(27) $$S_{9}\left(k_{r},k_{x}\right)=\exp\left\{-j\sqrt{k_{r}^{2}-k_{x}^{2}}\cos\theta_{e}\left(R_{e}-R_{ref}\right)-jk_{x}\sin\theta_{e}\left(R_{e}-R_{ref}\right)\right\}.$$

After bulk focusing, the residual phase at the reference range is removed. However, the residual phase of points not at the reference range remains. Moreover, the phase contains coupling terms between range and azimuth. For precise focusing of all points, the Stolt interpolation function is given as:

(28) $$k_{y}=\sqrt{k_{r}^{2}-k_{x}^{2}}\cos\theta_{e}+k_{x}\sin\theta_{e}.$$

After Stolt interpolation, the resampled signal becomes:

(29) $$S_{10}\left(k_{r},k_{x}\right)=\exp\left[-jk_{y}\left(R_{e}-R_{ref}\right)\right].$$

From Equation (29), it is evident that the coupling between range and azimuth has been removed. The phase is a linear function of $k_{y}$. Then, the inverse fast Fourier transform is implemented on Equation (29) to complete imaging.

**Figure 4:**
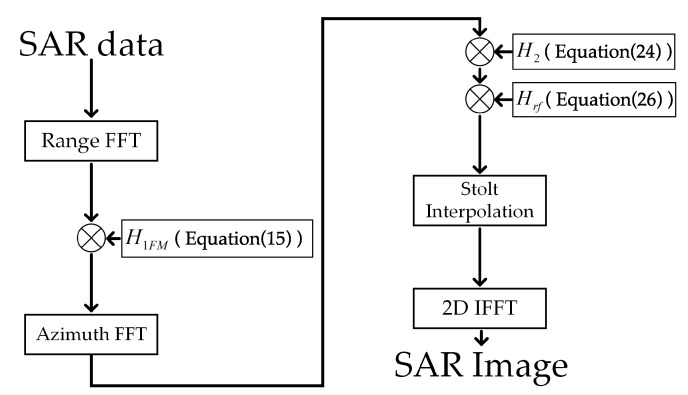
Flowchart of modified omega-K.

According to the analysis mentioned above, the whole imaging process is shown in Figure 4.

The specific steps are as follows:

(1) Performing range fast Fourier transform (FFT) on SAR data gets $S_{2}\left(f_{r},\eta \right)$.
(2) Multiplying Equation (15) and $S_{2}\left(f_{r},\eta \right)$ gets $S_{3}\left(f_{r},\eta \right)$.
(3) Performing azimuth fast Fourier transform (FFT) on $S_{3}\left(f_{r},\eta \right)$ gets $S_{7}\left(k_{r},k_{x}\right)$.
(4) Multiplying Equation (24) and $S_{7}\left(k_{r},k_{x}\right)$ gets $S_{8}\left(k_{r},k_{x}\right)$.
(5) Multiplying Equation (26) and $S_{8}\left(k_{r},k_{x}\right)$ gets $S_{9}\left(k_{r},k_{x}\right)$.
(6) Performing Stolt interpolation on $S_{9}\left(k_{r},k_{x}\right)$ gets $S_{10}\left(k_{r},k_{x}\right)$.
(7) Performing 2D-IFFT on $S_{10}\left(k_{r},k_{x}\right)$ gets output SAR focusing results.

## 4. Simulation Results

In this section, to demonstrate the effectiveness of the proposed imaging algorithm, experimental simulations of parallel bistatic forward-looking SAR are carried out. The system parameters are listed in Table 1. Four points at different locations were chosen to compare the imaging performance. They were $P_{0}\left(0,0\right)$, $P_{1}\left(0,500\right)$, $P_{2}\left(200,0\right)$, and $P_{3}\left(200,500\right)$. The unit of the coordinates is meters. The omega-K imaging algorithm based on the traditional three-parameters hyperbolic range model [13] was selected as the reference.

**Figure 5:**
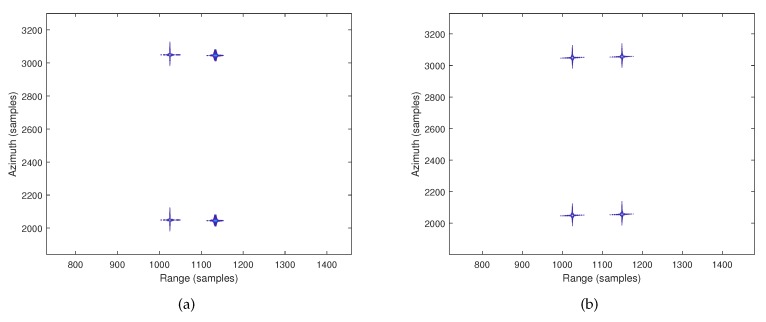
Imaging results. (**a**) Imaging results of the traditional hyperbolic omega-K algorithm. (**b**) Imaging results of the proposed hyperbolic omega-K algorithm.

Figure 5 is the comparison of the overall imaging performance before geometric correction. Figure 5a is the result of the traditional imaging algorithm, and Figure 5b is the result of the proposed imaging algorithm. In Figure 5a, although the four points can be successfully focused, the quality of the right two points has distortion. In contrast, Figure 5b shows that the proposed algorithm achieves a better focus quality on the right two points than the traditional algorithm.

**Figure 6:**
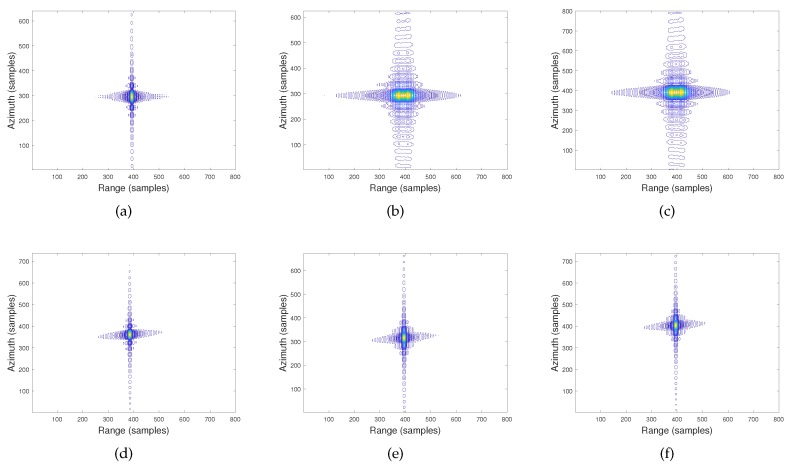
Imaging results. (**a**) Imaging result of $P_{0}$ by the traditional algorithm. (**b**) Imaging result of $P_{2}$ by the traditional algorithm. (**c**) Imaging result of $P_{3}$ by the traditional algorithm. (**d**) Imaging result of $P_{0}$ by the proposed algorithm. (**e**) Imaging result of $P_{2}$ by the proposed algorithm. (**f**) Imaging result of $P_{3}$ by the proposed algorithm.

**Figure 7:**
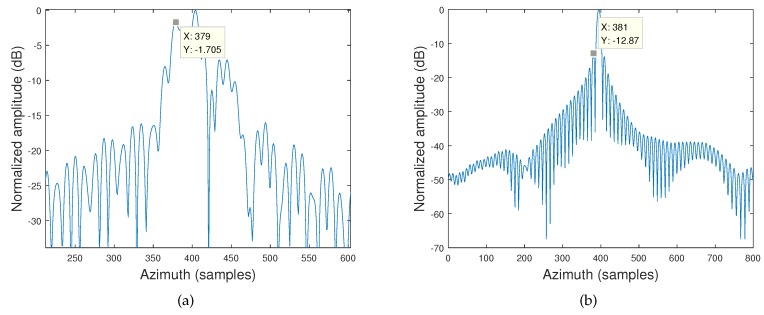
Azimuth impulse response of $P_{3}$. (**a**) Traditional hyperbolic omega-K algorithm. (**b**) Proposed hyperbolic omega-K algorithm.

To observe the imaging performance more intuitively, the sub-images of the four points extracted from Figure 5 are given by Figure 6. Figure 6a–c presents the imaging results of $P_{0},P_{2},\mathrm{and}P_{3}$ achieved by the traditional hyperbolic range model given in [13], respectively. Figure 6e,f shows the imaging quality of the three targets obtained by the proposed modified omega-K imaging algorithm. From Figure 6a,d, both algorithms can obtain an excellent focusing quality of the scene center $P_{0}$. For the omega-K algorithm, the scene center is always chosen as the reference point to perform bulk focusing. For the points away from the center ($P_{2}$ and $P_{3}$), it is evident that the proposed algorithm performs much better than the traditional algorithm. For further analysis, the azimuth impulse response of the farthest point $P_{3}$ is given in Figure 7. Table 2 gives out the peak sidelobe ratio (PSLR) and the integrated sidelobe ratio (ISLR) of targets $P_{3}$.

Figure 7a is achieved by the traditional hyperbolic omega-K algorithm. Figure 7b is achieved by the proposed hyperbolic omega-K algorithm. Compared with the traditional omega-K algorithm, the proposed omega-K algorithm can improve the performance of the azimuth impulse response. The objective image quality values demonstrated the effectiveness of the proposed omega-K algorithm.

## 5. Conclusions

In this article, an improved hyperbolic range model was proposed to deal with the particular form of the echo of bistatic forward-looking SAR. The modified omega-K imaging algorithm based on the hyperbolic range model was used to finish focusing. The high-order terms were taken into account to reduce the range approximation error. Extra phase compensation benefited the focusing of the omega-K algorithm. Compared with the range model without high-order compensation terms, the proposed method showed the effectiveness of imaging quality by simulation results.

## Figures and Tables

**Table 1:** Simulation parameters.

Parameters	Values	Parameters	Values
Carrier frequency	9 GHz	Transmitter center slant range	4300 m
Pulse duration	2 μs	Transmitter squint angle	$7^{\circ }$
Bandwidth	200 MHz	Receiver center slant range	3600 m
Sampling frequency	300 MHz	Receiver forward-looking angle	$33^{\circ }$
Pulse repetition frequency	1 kHz	Sensor speed	200 m/s

**Table 2:** Image quality parameters of $P_{3}$. PSLR, peak sidelobe ratio; ISLR, integrated sidelobe ratio.

Targets	PSLR (dB)		ISLR (dB)	
	Azimuth	Range	Azimuth	Range
Traditional omega-K algorithm	−1.705	-	-	-
Proposed omega-K algorithm	−12.87	−13.33	−8.86	−9.9558
